# Multiple serum biomarkers associate with mortality and interstitial lung disease progression in systemic sclerosis

**DOI:** 10.1093/rheumatology/keae110

**Published:** 2024-02-15

**Authors:** Matthew James Sinclair Parker, Adelle S Jee, Dylan Hansen, Susanna Proudman, Peter Youssef, Tony J Kenna, Wendy Stevens, Mandana Nikpour, Joanne Sahhar, Tamera J Corte

**Affiliations:** Faculty of Medicine and Health, The University of Sydney, Sydney, Australia; Rheumatology Department, RPA Institute for Academic Medicine, Royal Prince Alfred Hospital, Sydney, Australia; NHMRC Centre of Research Excellence in Pulmonary Fibrosis, Sydney, Australia; Faculty of Medicine and Health, The University of Sydney, Sydney, Australia; NHMRC Centre of Research Excellence in Pulmonary Fibrosis, Sydney, Australia; Department of Respiratory Medicine, Royal Prince Alfred Hospital, Sydney, Australia; Department of Rheumatology, St Vincent’s Hospital, Melbourne, Australia; Rheumatology Unit, Royal Adelaide Hospital, Adelaide, Australia; Discipline of Medicine, University of Adelaide, Adelaide, Australia; Faculty of Medicine and Health, The University of Sydney, Sydney, Australia; Rheumatology Department, RPA Institute for Academic Medicine, Royal Prince Alfred Hospital, Sydney, Australia; Institute for Musculoskeletal Health, Camperdown, Sydney, Australia; Centre for Immunology and Infection Control, School of Biomedical Sciences, Queensland University of Technology, Brisbane, Australia; Department of Rheumatology, St Vincent’s Hospital, Melbourne, Australia; Department of Rheumatology, St Vincent’s Hospital, Melbourne, Australia; Department of Medicine, The University of Melbourne, Melbourne, Australia; Department of Rheumatology, Monash Health, Melbourne, Australia; Department of Medicine, Central Clinical School, Monash University, Melbourne, Australia; Faculty of Medicine and Health, The University of Sydney, Sydney, Australia; NHMRC Centre of Research Excellence in Pulmonary Fibrosis, Sydney, Australia; Department of Respiratory Medicine, Royal Prince Alfred Hospital, Sydney, Australia

**Keywords:** scleroderma, prognosis, VCAM-1, E-selectin, SP-D, CXCL4, FVC

## Abstract

**Objectives:**

To investigate the prognostic utility of 28 serum biomarkers in systemic sclerosis (SSc), SSc-associated interstitial lung disease (SSc-ILD) and clinically relevant disease subgroups.

**Methods:**

Participants with sera, high-resolution CT and lung function within 12 months of baseline were identified from the Australian Scleroderma Cohort Study. Baseline was the time of serum collection. Twenty-seven of the prespecified 28 serum biomarkers were analysed and biomarker associations with mortality and ILD progression were investigated in univariable and multivariable analyses, including within disease subgroups and combined with established risk factors for poorer prognosis in SSc.

**Results:**

A total of 407 participants were identified, 252 (61.9%) with SSc-ILD. The median (interquartile range) follow-up after biomarker measurement was 6.31 (3.11–9.22) years. Sixteen biomarkers were associated with increased mortality. High levels of VCAM-1 were most strongly associated with mortality [hazard ratio (HR) 3.55; 95% CI 2.37–5.33; *P < *0.001]. Five additional biomarkers had an HR >2: SP-D (2.28, 1.57–3.31; *P < *0.001), E-selectin (2.19, 1.53–3.14; *P < *0.001), IL-6 (2.15, 1.50–3.09; *P < *0.001), MMP-3 (2.05, 1.42–2.95; *P < *0.001) and ET-1 (2.03, 1.40–2.92; *P < *0.001). Eleven biomarkers were independently associated with mortality following adjustment for sex, age and baseline forced vital capacity (FVC%predicted). Three biomarkers were associated with ILD progression at 1-year follow-up: CXCL4 (odds ratio 2.67, 1.46–4.88; *P* = 0.001), MMP-1 (2.56, 1.43–4.59; *P = *0.002) and ET-1 (2.18, 1.24–3.83; *P = *0.007).

**Conclusion:**

Multiple biomarkers, especially VCAM-1, E-selectin, SP-D and CXCL4, provide prognostic utility beyond that of established risk factors for patients with SSc.

Rheumatology key messages

- Multiple serum biomarkers provide prognostic utility beyond that of the previously established risk factors.
- VCAM-1 is strongly associated with mortality in patients with SSc.
- CXCL4 is associated with progression of SSc-associated interstitial lung disease in univariable analysis.

## Introduction

SSc is a multisystem autoimmune disease characterized by tissue and organ fibrosis, vasculopathy and inappropriate inflammation [1]. Patients with SSc have >200-fold higher mortality compared with healthy individuals. Disappointingly, despite improvements in management, overall survival has only marginally improved over the last few decades [2, 3].

A major contributor to mortality in patients with SSc is interstitial lung disease (ILD), present in up to 80% of patients with SSc [3, 4]. Not all patients with SSc-associated ILD (SSc-ILD) will have clinically significant ILD and there is wide variation in the disease course for SSc-ILD [5]. Some patients may have stable disease over extended timeframes even without specific therapy, while others have inexorable progression despite optimal therapy, leading to respiratory failure and death. As potential therapeutic options for SSc improve but generally only attenuate progression of disease, accurate prognostication for the individual patient has never been more important.

Clinical and demographic prognostic risk factors for SSc-ILD include male sex, age and lower forced vital capacity (FVC) [6]. Alongside these, several serum biomarkers have been studied with some success [7]. However, to date, no biomarker or combination of biomarkers has been validated across multiple clinical cohorts to predict prognosis in SSc-ILD [8].

In our earlier work, we performed a comprehensive literature review of serum biomarkers in SSc-ILD and identified 28 biomarkers for further study and have evaluated these biomarkers for the identification of SSc-ILD [9, 10]. In the present study, our primary objective was to evaluate the prognostic utility of these 28 serum biomarkers in patients with SSc and SSc-ILD, alone and in combination with established clinical and demographic risk factors. Pre-specified subgroups including limited and dcSSc, patients with limited or extensive ILD at baseline, early disease and the presence of pulmonary hypertension at baseline, were identified for subgroup analysis.

## Methods

### Participants

The Australian Scleroderma Cohort Study (ASCS) is a national, multicentre, longitudinal observational cohort established in 2007 by the Australian Scleroderma Interest Group (ASIG). All Australian physicians are invited to refer SSc patients to 12 national screening centres. Patients fulfilling ACR/EULAR classification for SSc are eligible for enrolment [11]. Participants provide written consent at recruitment. Utilizing a standardized protocol, comprehensive de-identified data are recorded on consecutive patients at baseline and every 12 months including clinical assessment, investigations, treatment and outcomes, including vitality and lung transplantation. High resolution CT (HRCT) scan of the chest is performed when clinically indicated according to physician judgement. Disease extent on HRCT, defined according to the method of Goh *et al.* [12], is available on a subset of patients from prior studies. Selected centres have baseline and serial serum samples stored in a linked biobank. Clinical management remains as per the treating physician.

Study participants were identified through the ASCS. All patients with sera, HRCT to confirm presence or absence of ILD and lung function within 12 months of baseline at the time of study commencement (1 February 2017) were included. SSc-ILD was defined as SSc with radiologically confirmed ILD on HRCT. Patients with insufficient data to confirm ILD status or serum were excluded.

Our study complies with the Declaration of Helsinki. The Australian Scleroderma Cohort Study has human research ethics approval from all participating centres. Approval for this study was granted by the Research Ethics and Governance Office (protocol numbers X16-0311 & LNR/16/RPAH/406 RPAH zone, SVHM HREC LRR 0121/20). Written informed consent was obtained from all participants.

### Definitions

Baseline was defined as the time of serum collection. Disease duration was defined as time from first non-Raynaud symptom until baseline. Percentage predicted values for forced vital capacity (FVC%) were reported using the *Quanjer* Global Lung Initiative 2012 predictive equation [13]. ILD extent was defined as per Goh *et al.* with ‘limited disease’ considered for <20% involvement on HRCT and ‘extensive disease’ for >20% involvement [12]. Disease subgroup (limited or diffuse) was defined by enrolling physician. In the absence of a widely accepted specific definition, early disease was defined as <5 years from first non-Raynaud’s disease manifestation to biomarker collection.

Pulmonary arterial hypertension (PAH) was defined using right heart catheter–measured haemodynamic criteria applying 2019 ESC/ERS criteria. In a small proportion of participants where peripheral vascular resistance was not recorded the pre-existing definition of PAH was applied [14, 15]. Mortality was considered as all-cause mortality. ILD progression was defined as a relative decline in FVC of ≥10% from baseline FVC [8]. Follow-up data were censored on 1 May 2019.

### Serum samples and biomarker measurement

Serum samples were obtained from the ASCS biobanks and stored at –80°C until analysis. Sera with more than two freeze–thaw cycles were excluded. Twenty-eight biomarkers were selected by comprehensive literature review [9]. Equipment required to measure KL-6 was unavailable. The remaining 27 biomarkers were analysed at a single laboratory following manufacturer’s protocols by magnetic Luminex (SP-D, MMP-1, MMP-3, MMP-7, MMP-12, TIMP-1, Ca15-3, periostin, CCL-2, IL-6, VEGF, IL-8, CXCL10, CXCL12, CXCL13, E-selectin, CXCL4, CCL-18, ICAM-1, VCAM-1), bead-based assay (TGF-β1, -β2, -β3) and ELISA (amphiregulin, fibulin-1, LOXL-2, ET-1). Non-abbreviated biomarker labels and assay details are shown in Supplementary Table S1 (available at *Rheumatology* online). Any biomarkers with >25% of results below detectable range in initial analysis were re-tested with higher sensitivity assays. Individuals with undetectable biomarker values were imputed at half the lower limit of detection. Individual results above the quantitative threshold were imputed at maximum quantifiable value.

### Statistical analysis

Continuous variables are reported as mean (s.d.) or median [interquartile range (IQR)] as appropriate, and categorical variables as an absolute number (relative frequency). *P*-values <0.05 were considered statistically significant.

Biomarker values were dichotomized with threshold values (in pg/ml) calculated using the Liu method of optimal cut-point estimation for a diagnostic test for the relevant outcome. Biomarker outcome associations were studied in univariable and multivariable analysis. In multivariable analysis, biomarkers were analysed with gender, age and baseline FVC%. The results of these analyses are expressed as a hazard ratio (HR) of outcome with 95% CI and *P*-value.

All statistical analysis was performed using Stata statistical software (StataCorp. 2023. Stata Statistical Software: Release 15, College Station, TX: StataCorp LLC).

## Results

Of 407 SSc participants identified, 252 (61.9%) had SSc-ILD. Patients with SSc-ILD had a median age of 58.7 years (IQR 50.6–66.7), 202 (80.2%) were female, mean FVC% was 87.5% (±21.9%) with a median follow-up 6.50 years (IQR 2.9–9.2). Demographic and selected clinical features are detailed in Table 1. Participants with ILD were more likely to be non-Caucasian, have diffuse disease (39.7%), be anti-Scl70 positive (35.6%) and anti-centromere negative (19.6% positive), have a lower baseline FVC% and increased mortality (see Supplementary Fig. S1, available at *Rheumatology* online). HRCT data to establish ILD extent (‘limited’ or ‘extensive’ as defined in methods) was only available for 197/252 (78.2%) participants.

**Table 1.** Participant characteristics

Variable		SSc (*n* = 407)	SSc-ILD (*n* = 252)	SSc No-ILD (*n* = 155)	*P*-value
Age at biomarker collection		58.81 (50.51–66.40)	58.67 (50.56–66.69)	59.24 (49.46–65.51)	0.719
Disease duration, years		8.29 (3.38–16.98)	6.83 (2.52–15.63)	7.65 (2.43–15.58)	0.742
Follow-up period, years		6.31 (3.11–9.22)	6.50 (2.85–9.16)	6.09 (3.56–9.45)	0.699
Sex, *n* (%)	Male	80 (19.7)	50 (19.8)	30 (19.4)	0.905
	Female	327 (80.3)	202 (80.2)	125 (80.6)	
Caucasian, *n* (%)	No	45 (11.1)	37 (14.7)	8 (5.2)	**0.003**
	Yes	361 (88.9)	215 (85.3)	146 (94.8)	
Disease subtype, *n* (%)	Limited	272 (66.8)	152 (60.3)	120 (77.4)	**<0.001**
	Diffuse	135 (33.2)	100 (39.7)	35 (22.6)	
Antibody profile, *n* (%)	ANA positive	386 (94.8)	242 (96.0)	144 (92.9)	0.166
	Centromere	124 (30.7)	49 (19.6)	75 (48.7)	**<0.001**
	Scl-70	105 (26.1)	89 (35.6)	16 (10.5)	**<0.001**
	RNA Polymerase III	49 (15.2)	31 (15.3)	18 (15.1)	0.972
FVC%		92.4 (22.60)	87.5 (21.88)	100.4 (21.5)	**<0.001**
PAH, *n* (%)		70 (17.2)	42 (16.7)	28 (18.1)	0.717
Died during follow-up, *n* (%)		120 (29.5)	85 (33.7)	35 (22.6)	**0.017**
FVC% drop >10% during follow-up, *n* (%)		281 (72.6)	178 (75.4)		

Bold typeface used for statistically significant results (*P* ≤ 0.05).

SSc-ILD: SSc-associated interstitial lung disease; FVC%: forced vital capacity percentage predicted; PAH: pulmonary arterial hypertension.

### Mortality in entire SSc cohort

In univariable analysis of all participants, 16 biomarkers were associated with increased mortality (Table 2). High levels of VCAM-1 were most strongly associated with mortality (HR 3.55, 95% CI 2.37–5.33; *P* < 0.001). A further five biomarkers had a HR above 2.0, including SP-D (HR 2.28, 1.57–3.31; *P < *0.001), E-selectin (HR 2.19, 1.53–3.14; *P < *0.001), IL-6 (HR 2.15, 1.50–3.09; *P < *0.001), MMP-3 (HR 2.05, 1.42–2.95; *P < *0.001) and ET-1 (HR 2.03, 1.40–2.92; *P < *0.001).

**Table 2.** Biomarkers associated with mortality in univariable analysis

Biomarkers	SSc-Total (*n* = 407)		SSc-ILD (*n* = 252)		SSc no-ILD (*n* = 155)	
	Threshold (pg/mL)	HR (95% CI), *P*-value	Threshold (pg/mL)	HR (95% CI), *P*-value	Threshold (pg/mL)	HR (95% CI), *P*-value
VCAM-1	1 176 536.8	**3.55** (2.37–5.33), *P < *0.001	1103174.7	**4.04** (2.37–6.90), *P < *0.001	1203068.7	**3.99** (1.90–8.37), *P < *0.001
SP-D	33 023.6	**2.28** (1.57–3.31), *P < *0.001	32894.5	**2.06** (1.29–3.27), *P = *0.002	22099	**2.85** (1.37–5.95), *P = *0.005
E-selectin	40 267.0	**2.19** (1.53–3.14), *P < *0.001	40267.0	**2.22** (1.44–3.43), *P < *0.001	37989.1	**2.06** (1.05–4.04), *P = *0.036
IL-6	4.3	**2.15** (1.50–3.09), *P < *0.001	4.4	**2.08** (1.36–3.19), *P = *0.001	3.55	**2.04** (1.04–3.99), *P = *0.037
MMP-3	14 226.4	**2.05** (1.42–2.95), *P < *0.001	14526.5	**2.36** (1.52–3.68), *P < *0.001	NS	NS
ET-1	11.8	**2.03** (1.40–2.92), *P < *0.001	10.6	**1.67** (1.08–2.58), *P = *0.022	13.0	**3.42** (1.67–7.02), *P = *0.001
MMP-7	2069.5	**1.99** (1.39–2.85), *P < *0.001	2281.7	**1.92** (1.25–2.96), *P = *0.003	1753.1	**2.22** (1.13–4.34), *P = *0.020
CXCL12	482.6	**1.90** (1.32–2.73), *P = *0.001	485.7	**2.18** (1.41–3.35), *P < *0.001	682.55	**4.23** (1.95–9.17), *P < *0.001
CXCL13	81.0	**1.88** (1.31–2.70), *P = *0.001	80.5	**1.85** (1.20–2.86), *P = *0.005	96.6	**2.01** (1.03–3.92), *P = *0.040
MMP-1	2256.5	**1.68** (1.17–2.42), *P = *0.005	2285.5	**1.70** (1.10–2.62), *P = *0.016	NS	NS
CCL-18	85 515.0	**1.61** (1.12–2.30), *P = *0.009	NS	NS	81596.766	**2.03** (1.04–3.97), *P = *0.037
CA15-3	49.6	**1.56** (1.09–2.24), *P = *0.015	51.0	**1.67** (1.09–2.58), *P = *0.019	NS	NS
CCL-2	534.4	**1.56** (1.08–2.24), *P = *0.018	NS	NS	NS	NS
VEGF	99.4	**1.56** (1.07–2.27), *P = *0.021	NS	NS	NS	NS
Periostin	173 525.6	**1.55** (1.08–2.22), *P = *0.017	174842.5	**1.70** (1.11–2.61), *P = *0.016	NS	NS
CXCL/IL-8	19.8	**1.47** (1.02–2.10), *P = *0.036	NS	NS	NS	NS
Age^a^		**1.06** (1.04–1.08), *P < *0.001		**1.06** (1.04–1.08), *P < *0.001		**1.06** (1.03–1.10), *P < *0.001
Sex (male)		**2.11** (1.43–3.11), *P < *0.001		**2.62** (1.67–4.12), *P < *0.001		**1.27** (0.58–2.81), *P = *0.55
FVC% baseline^b^		**0.98** (0.97–0.99), *P < *0.001		**0.98** (0.97–0.99), *P < *0.001		**0.99** (0.97–1.00), *P = *1.27

Bold typeface used for statistically significant results (*P* ≤ 0.05).

a Per year increase.

b Per percentage point increase. SSc-ILD: SSc-associated interstitial lung disease; SSc no-ILD: SSC, no interstitial lung disease; FVC%: forced vital capacity percentage predicted; NS: not significant.

In multivariable analysis, combined with age, sex and baseline FVC%, 11 biomarkers remained significantly associated with increased mortality (Table 3). The strongest biomarker association with mortality was VCAM-1 (HR 2.85, 1.86–4.38; *P < *0.001).

**Table 3.** Biomarkers associated with mortality in multivariable analysis combined with gender, age and baseline FVC%

Biomarker	SSc-Total (*n* = 407) HR (95% CI), *P*-value	SSc-ILD (*n* = 252) HR (95% CI), *P*-value	SSc no-ILD (*n* = 155) HR (95% CI), *P*-value
VCAM-1	**2.85** (1.86–4.38), *P < *0.001	**3.11** (1.77–5.46), *P < *0.001	**3.61** (1.66–7.86), *P = *0.001
E-selectin	**2.20** (1.51–3.20), *P < *0.001	**2.29** (1.46–3.61), *P < *0.001	**2.03** (1.03–4.03), *P = *0.042
SP-D	**1.94** (1.33–2.83), *P = *0.001	**1.81** (1.13–2.89), *P = *0.014	**2.59** (1.24–5.43), *P = *0.012
ET-1	**1.82** (1.25–2.65), *P = *0.002	NS	**3.11** (1.51–6.42), *P = *0.002
CXCL12	**1.75** (1.21–2.54), *P = *0.003	**1.89** (1.20–2.95), *P = *0.006	**4.58** (1.98–10.58), *P < *0.001
CCL-18	**1.66** (1.15–2.39), *P = *0.006	NS	**2.01** (1.03–3.95), *P = *0.042
IL-6	**1.65** (1.12–2.42), *P = *0.010	NS	**2.02** (1.01–4.02), *P = *0.046
MMP-3	**1.65** (1.12–2.42), *P = *0.010	**1.79** (1.12–2.86), *P = *0.015	NS
MMP-7	**1.62** (1.12–2.35), *P = *0.010	NS	**2.05** (1.03–4.08), *P = *0.040
Ca15-3	**1.56** (1.08–2.24), *P = *0.018	**1.71** (1.10–2.65), *P = *0.017	NS
MMP-1	**1.54** (1.06–2.22), *P = *0.023	NS	NS
CXCL4	NS	**1.87** (1.20–2.92), *P = *0.005	NS

Bold typeface used for statistically significant results (*P* ≤ 0.05). SSc-ILD, SSc-associated interstitial lung disease; SSc no-ILD, SSc, no interstitial lung disease; NS, not significant; FVC%, forced vital capacity percentage predicted.

### Mortality in SSc-ILD and SSc no-ILD groups

In SSc-ILD participants, 12 biomarkers were associated with increased mortality on univariable analysis (Table 2). VCAM-1 had the strongest association (HR 4.04, 2.37–6.90; *P < *0.001). In multivariable analysis, seven biomarkers remained significant after adjusting for age, sex and baseline FVC% (Table 3). VCAM-1 and E-selectin had the strongest associations with mortality. Interestingly, CXCL4, which was not found to be associated with mortality on multivariable analysis in the total cohort or the SSc no-ILD group, predicted mortality in this SSc-ILD cohort.

In the SSc no-ILD group, nine biomarkers were associated with mortality on univariable analysis (Table 2). The strongest associations were with CXCL12 (HR 4.23, 1.95–9.17; *P < *0.001) and VCAM-1 (HR 3.99, 1.90–8.37; *P < *0.001). Interestingly, ET-1 (3.42, 1.67–7.02; *P = *0.001) had a stronger association with mortality in this group without ILD at baseline. CCL-18 was associated with mortality in this group but not in participants with ILD at baseline. In multivariable analysis (Table 3), the strongest association with mortality was again with CXCL12 followed by VCAM-1.

### Subgroup analyses

We performed univariable and multivariable survival analyses in pre-specified subgroups including early disease, disease subtype (diffuse and limited), ILD extent (limited and extensive ILD on baseline HRCT) and the presence or absence of PAH. The univariable Cox regression analyses are presented in the Supplementary material (Supplementary Table S2, available at *Rheumatology* online). The multivariable analyses, adjusting for age, sex and baseline FVC%, are shown in Table 4.

**Table 4.** Biomarkers associated with mortality in multivariable subgroup analysis

Subgroup		Biomarker	Adjusted HR (95% CI), *P*-value
Early disease (*n* = 136)		VCAM-1	**4.99** (1.85–13.44), *P = *0.001
		E-selectin	**3.95** (1.78–8.77), *P = *0.001
		CCL-2	**3.60** (1.55–8.35), *P = *0.003
		ET-1	**3.14** (1.08–9.13, *P = *0.035)
		CXCL12	**3.08** (1.40–6.78), *P = *0.005
		CCL-18	**2.82** (1.24–6.45), *P = *0.014
		MMP-1	**2.73** (1.28–5.81), *P = *0.009
		TIMP-1	**2.58** (1.16–5.72), *P = *0.020
		CXCL/IL-8	**2.31** (1.07–5.02), *P = *0.034
Disease subtype	Diffuse disease, *n* = 135	TIMP-1	**3.59** (1.77–7.29), *P < *0.001
		VCAM-1	**2.99** (1.41–6.36), *P = *0.004
		E-selectin	**2.87** (1.43–5.75), *P = *0.003
		Ca15-3	**2.42** (1.24–4.75), *P = *0.010
		SP-D	**2.41** (1.20–4.82), *P = *0.013
		CCL-18	**2.19** (1.14–4.21), *P = *0.019
		CXCL4	**2.08** (1.10–3.93), *P = *0.024
		MMP-1	**2.01** (1.03–3.92), *P = *0.042
	Limited disease, *n* = 272	VCAM-1	**2.89** (1.71–4.89), *P < *0.001
		MMP-3	**2.42** (1.52–3.86), *P < *0.001
		IL-6	**2.25** (1.41–3.60), *P = *0.001
		ET-1	**2.10** (1.31–3.35), *P = *0.002
		MMP-7	**2.04** (1.27–3.26), *P = *0.003
		E-selectin	**2.01** (1.26–3.19), *P = *0.003
		SP-D	**1.96** (1.21–3.19), *P = *0.006
		CXCL12	**1.78** (1.14–2.79), *P = *0.011
		CXCL13	**1.66** (1.05–2.64), *P = *0.031
ILD extent	ILD extent <20%, *n* = 111	VCAM-1	**2.79** (1.16–6.71), *P = *0.022
		E-selectin	**2.41** (1.16–5.03), *P = *0.019
		CXCL12	**2.40** (1.14–5.06), *P = *0.021
		IL-6	**2.27** (1.06–4.86), *P = *0.034
	ILD extent >20%, *n* = 86	SP-D	**2.58** (1.39–4.76), *P = *0.003
		CCL-18	**2.48** (1.29–4.77), *P = *0.007
		MMP-1	**2.25** (1.18–4.29), *P = *0.014
		Amphiregulin	**2.16** (1.12–4.19), *P = *0.022
PAH^a^	Yes, *n* = 70	SP-D	**2.64** (1.27–5.47), *P = *0.009
		E-selectin	**2.30** (1.18–4.48), *P = *0.014
	No, *n* = 337	VCAM-1	**2.86** (1.66–4.90), *P < *0.001
		CCL-18	**1.86** (1.17–2.97), *P = *0.009
		E-selectin	**1.81** (1.12–2.91), *P = *0.015
		MMP-3	**1.75** (1.07–2.84), *P = *0.024
		CXCL12	**1.75** (1.08–2.81), *P = *0.022
		CXCL4	**1.59** (1.00–2.54), *P = *0.050

Bold typeface used for statistically significant results (*P* ≤ 0.05).

a PAH was not investigated for with right heart catheter study in every patient in this cohort. ILD: interstitial lung disease; PAH: pulmonary arterial hypertension.

Elevated VCAM-1 had the strongest association with increased mortality across all subgroups, with the exception of patients with extensive ILD at baseline and those with PAH. E-selectin also was associated with mortality across all subgroups except in participants with extensive ILD at baseline.

CXCL12 was associated with mortality in participants with early disease, limited disease, limited extent ILD and without PAH. In patients with more extensive ILD at baseline, SP-D, CCL-18, MMP-1 and Amphiregulin were associated with increased mortality.

### Progression of lung disease in SSc-ILD

In the 252 participants with SSc-ILD, CXCL4 (HR 1.38, 1.2–1.88; *P = *0.039) was associated with ILD progression over the entire study period, on univariable analysis. This association did not remain significant when adjusted for sex, age and baseline FVC%.

Three biomarkers were associated with ILD progression at 1 year: CXCL4 [odds ratio (OR) 2.67, 1.46–4.88, 1.67; *P = *0.001], MMP-1 (OR 2.56, 1.43–4.59; *P = *0.002) and ET-1 (OR 2.18, 1.24–3.83; *P = *0.007). The relationship of biomarkers and ILD progression in the pre-specified subgroups are shown in Table 5. E-selectin was negatively associated with ILD progression in participants with limited disease (HR 0.68, 0.47–1.00; *P = *0.049) and PAH (HR 0.42, 0.20–0.90; *P = *0.025).

**Table 5.** Biomarkers associated with ILD progression in univariable subgroup analysis

Subgroup			Biomarker: HR (95% CI), *P*-value
Early disease	*n* = 87		MMP-3: **1.89** (1.08–3.33), *P = *0.027
			CCL-18: **1.79** (1.07–3.00), *P = *0.028
Disease subtype		Diffuse disease, *n* = 100	E-selectin: **2.18** (1.19–4.01), *P = *0.012
			ICAM-1: **2.03** (1.23–3.36), *P = *0.006
			LOXL2: **1.83** (1.14–2.94), *P = *0.012
		Limited disease, *n* = 152	CXCL4: **1.84** (1.12–3.01), *P = *0.016
			Ca15-3: **1.51** (1.02–2.23), *P = *0.038
			E-selectin: **0.68** (0.47–1.00), *P = *0.049
			ICAM-1: **0.64** (0.44–0.93), *P = *0.019
ILD extent		ILD extent <20%, *n* = 111	MMP-7: **2.06** (1.20–3.54), *P = *0.009
		ILD extent >20%, *n* = 86	CXCL4: **1.92** (1.13–3.28), *P = *0.016
			ET-1: **1.83** (1.06–3.13), *P = *0.029
			Ca15-3: **1.76** (1.03–3.00), *P = *0.039
			CCL-18: **1.72** (1.02–2.89), *P = *0.040
PAH^a^		Yes, *n* = 42	CXCL4: **3.97** (1.21–13.05), *P = *0.023
			MMP-1: **2.01** (1.00–4.03), *P = *0.050
			E-selectin: **0.42** (0.20–0.90), *P = *0.025
		No, *n* = 210	N/A

Bold typeface used for statistically significant results (*P* ≤ 0.05).

a PAH was not investigated for with right heart catheter study in every patient in this cohort. ILD: interstitial lung disease; PAH: pulmonary arterial hypertension.

## Discussion

This study describes the largest panel of biomarkers measured concurrently in a national, well-phenotyped cohort of patients with SSc. All the biomarkers studied had been previously demonstrated to have associations with potentially clinically meaningful outcomes. In this study, we analysed the biomarkers collectively and with established risk factors to determine which biomarker individually contributes most powerfully to the outcomes of interest. Accurate prognostication may help inform management strategies for screening, treatment interventions and intensity.

The most striking association in this study was between increased mortality and elevated levels of VCAM-1. VCAM-1 demonstrated a strong association with mortality in participants both with and without ILD, maintained when accounting for established risk factors for worse prognosis, age, gender and FVC%. This relationship remained strong for participants both with and without ILD at baseline as well as in clinically relevant subgroups, except participants with extensive ILD and PAH. This would suggest that elevated VCAM-1 may be an important prognostic marker in patients with SSc in general. The weaker association in participants with extensive ILD and PAH was interesting as these subgroups of patients tend to have higher mortality. Possible explanations for this finding include that VCAM-1 may be a marker of progression independent of ILD and PAH disease manifestations, or that VCAM-1 may predict progression prior to these manifestations which, when established, progress in association with other biomarkers.

VCAM-1 is a 90-kDa glycoprotein predominantly expressed in endothelial cells with expression upregulated by pro-inflammatory cytokines such as TNF-α. It is a transmembrane protein involved in the adhesion and trans-endothelial migration of leucocytes. In some diseases, VCAM-1 is also expressed on tissue macrophages, dendritic cells and other cell types potentially relevant to SSc pathophysiology [16]. Elevated VCAM-1 has previously been associated with mortality in SSc and clinical progression in SSc [17] and idiopathic pulmonary fibrosis [18].

Two additional biomarkers, E-selectin and SP-D, were consistently associated with mortality in our study. E-selectin was associated with mortality on univariable and multivariable analysis in all subgroups, except for participants with extensive ILD. E-selectin is a cell adhesion molecule expressed solely by endothelial cells. Levels have been found to be high in patients with SSc [19]. E-selectin has been correlated with disease activity in SSc and systemic organ involvement, particularly renal crisis [20, 21].

SP-D similarly was associated with mortality including in all subgroups except in early disease, limited extent ILD and in patients without PAH. SP-D may predict progression in participants who have already developed major internal organ manifestations.

SP-D is a pulmonary surfactant lipoprotein and elevated serum levels have been found to reflect the extent of damage to the capillary/epithelial barrier in ILD [22]. SP-D levels have been found to be elevated in patients with SSc-ILD [23] and may reflect the severity of SSc-ILD [24–27]. Levels of SP-D have been found to be higher in patients with SSc-ILD compared with those without ILD [10, 24, 28]. In a large prospective cohort, SP-D values combined with anti-Scl-70 (topoisomerase I) antibodies were used to accurately identify patients with SSc-ILD [28] but SP-D levels were not correlated with the severity of lung disease, its progression or mortality. Our data suggests there is an important correlation between mortality and elevated SP-D levels.

Other biomarker associations appear to be more specific for certain participant subgroups. MMP-3 was only associated with increased mortality in participants with ILD at baseline and in those with limited disease. Amphiregulin was only associated with mortality in those with extensive ILD at baseline. The association between CXCL12 and mortality was particularly strong in participants without ILD at baseline, where it was the strongest single biomarker association. Elevated levels of CXCL12 (or elevated cellular expression of the corresponding receptor CXCR4) have been found in previous studies of patients with SSc, but there are no previous reports of a link to mortality or of disproportionate expression in particular disease subgroups [29–31]. Genetic polymorphisms in the *SDF1* gene that would be predicted to result in increased levels of CXCL12 have been found to be more common in SSc patients with PAH and/or digital ulcers [32]. ET-1 was particularly associated with mortality in participants with early and limited disease.

The associations between biomarkers and mortality were strongest in participants with early disease. This may in part link to the natural history of the disease where patients with longer established disease are less likely to progress. Additionally, in patients with more severe ILD, lower FVC% is a powerful clinical predictor, and so it is difficult to demonstrate any additional prognostic benefit beyond FVC% in this cohort. We have demonstrated that multiple serum biomarkers improve prognostic accuracy beyond that of established risk factors. This is particularly relevant in this group of patients with early disease—knowing early which patients are likely to do worse could influence treatment decisions and hopefully help better target therapies to those patients who are most likely to benefit.

Collectively our data suggests that multiple biomarkers could add to established risk factors in better predicting mortality in patients with a broad spectrum of SSc. It may be a that a combination of biomarkers including some that are useful in all patients combined with others more specific to the individual phenotype will provide the best performance.

Our study demonstrated potentially important associations between CXCL4 and ILD disease progression, as defined by decline in FVC%. CXCL4 was associated with progression in participants with ILD at baseline on univariable analysis. CXCL4 is released predominantly from activated platelets and has a chemotactic activity on neutrophils, monocytes and fibroblasts. It also stimulates pro-fibrotic cytokines and inhibits the expression of antifibrotic IFN-γ [33]. A proteome-wide study in SSc found that plasmacytoid dendritic cells predominantly secrete CXCL4 [34]. CXCL4 levels were markedly elevated in SSc patients and highly correlated with skin fibrosis, ILD and PAH [34]. Elevated CXCL4 levels have also been shown to be associated with a more rapid decline in gas transfer and decreased levels were predictive of improved pulmonary function during immunosuppressive therapy [34]. CXCL4 overexpression in SSc has a direct mechanistic link to upregulated IFN-1 pathways by acting as both a chaperone and adjuvant in breaking immune tolerance to self-DNA [35]. The findings in our study contribute to and support this emerging literature.

Our results should be interpreted in the context of the patient population. Although representative of SSc populations seen in clinical practice, the relatively high proportion of long-established and limited disease may have influenced the results in ways for which we were unable to control. PAH, an important contributor to increased mortality, was defined accurately by strict criteria but not all patients were systematically investigated for its presence. This was a retrospective study and biospecimens were collected at variable times in relation to definitions of disease subgroups. No clinically correlated externally validated values for the biomarkers are available so the thresholds calculated in this study are specific to this cohort and the techniques described. Participant characteristics and subgroups were defined at baseline and not assessed during follow-up. This study did not look at cause of death which could be particularly important in those with strong biomarker signals. In addition to the variables already described, anti-Scl70 has also been associated with progression of SSc-ILD [34]. In *post hoc* analysis (data not shown but available on request), adding anti-Scl70 status to multivariable analysis had no significant effect on the serum biomarker associations.

The strengths of our study include the unique combination of a large, well-phenotyped cohort of patients with comprehensive datasets and long follow-up together with the largest multiple biomarker panel investigated in SSc research to date. Further work could legitimately focus on certain subgroups in more detail, such as those with early diffuse disease or in those with already more extensive ILD at baseline. It would be useful to see how biomarkers can be best combined together and with clinical variables to improve prognostication. External validation of our findings is required. The strong associations identified in this study between identified biomarkers and important prognostic outcomes may support ongoing research into disease pathogenesis and management.

### Conclusion

We have demonstrated that multiple biomarkers, especially VCAM-1, E-selectin, SP-D and CXCL4, provide prognostic utility beyond that of established risk factors for patients with SSc. These findings provide a framework for future investigation.

## Supplementary Material

keae110_Supplementary_Data

## Data Availability

The data underlying this article cannot be shared publicly to preserve the privacy of individuals that participated in the study. The data will be shared on reasonable request to the corresponding author.
