# Supplementary material for: Multiple serum biomarkers associate with mortality and interstitial lung disease progression in systemic sclerosis
Source: Rheumatology (Oxford). 2024 Feb 15;63(11):2981–8. doi: 10.1093/rheumatology/keae110 (PMC11534140; doi:10.1093/rheumatology/keae110)
Supplement: keae110_Supplementary_Data [file keae110_supplementary_data.docx]

**Supplemental Material:**

| Platform | Biomarker | Complete label | Supplier | Catalogue number |
| --- | --- | --- | --- | --- |
| Luminex | SP-D | Surfactant protein D | R&D | LXSAHM-15 |
| Luminex | MMP-3 | Matrix metalloproteinase-3 | R&D | LXSAHM-15 |
| Luminex | MMP-12 | Matrix metalloproteinase-12 | R&D | LXSAHM-15 |
| Luminex | MMP-7 | Matrix metalloproteinase-7 | R&D | LXSAHM-15 |
| Luminex | MMP-1 | Matrix metalloproteinase-1 | R&D | LXSAHM-15 |
| Luminex | Periostin | — | R&D | LXSAHM-15 |
| Luminex | CXCL-13 | C-X-C motif chemokine ligand-13 | R&D | LXSAHM-15 |
| Luminex | CCL-2 (MCP-1) | Chemokine ligand 2 (monocyte chemoattractant protein-1) | R&D | LXSAHM-15 |
| Luminex | IL-6 | Interleukin-6 | R&D | LXSAHM-15 |
| Luminex | VEGF | Vascular endothelial growth factor | R&D | LXSAHM-15 |
| Luminex | IL-8 (CXCL-8) | Interleukin-8 (C-X-C motif chemokine ligand-8) | R&D | LXSAHM-15 |
| Luminex | CXCL-10 (IFNγ induced protein 10) | C-X-C motif chemokine ligand-10 (Interferon γ induced protein 10) | R&D | LXSAHM-15 |
| Luminex | CXCL-12 | C-X-C motif chemokine ligand-12 | R&D | LXSAHM-15 |
| Luminex | E-selectin | — | R&D | LXSAHM-15 |
| Luminex | CXCL-4 | C-X-C motif chemokine ligand-4 | R&D | LXSAHM-01 |
| Luminex | TIMP-1 | Tissue inhibitor of metalloproteinase-1 | R&D | LXSAHM-03 |
| Luminex | Ca 15-3 |  | R&D | LXSAHM-03 |
| Luminex | CCL-18 | Chemokine ligand-18 | R&D | LXSAHM-03 |
| Luminex | ICAM-1 | Intercellular adhesion molecule 1 | R&D | LXSAHM-02 |
| Luminex | VCAM-1 | Vascular cell adhesion molecule 1 | R&D | LXSAHM-02 |
| Magnetic bead | TGF-β1/-2/-3 | Transforming growth factor-β1, -2, -3 | Millipore | TGFBMAG-64K-03 |
| ELISA | Amphiregulin | —— | R&D | RDSDAR00 |
| ELISA | KL-6 | Krebs von den Lungen-6 | Abbexa | abx350080 |
| ELISA | Fibulin-1 | — | Abbexa | abx350481 |
| ELISA | LOXL-2 | Lysyl osidase-like 2 | Biomatik | EKC34510 |
| ELISA | ET-1 | Endothelin 1 | R&D | RDSDET-100 |

**Supplementary Table S1: Serum biomarkers analysed and assays used.**

| Subgroup |  | Biomarker | HR (CI; p-value) |
| --- | --- | --- | --- |
| Early disease (n=136) | | **VCAM-1** | 6.57 (2.67-16.19), p<0.001 |
|  |  | **CXCL12** | 4.32 (2.12-8.80), p<0.001 |
|  |  | **MMP-1** | 3.58 (1.77-7.21), p<0.001 |
|  |  | **ET1** | 3.34 (1.29-8.68), p=0.013 |
|  |  | **E-Selectin** | 3.03 (1.50-6.12), p=0.002 |
|  |  | **CCL2** | 2.77 (1.33-5.79), p=0.007 |
|  |  | **MMP-7** | 2.76 (1.36-5.61), p=0.005 |
|  |  | **CXCL/IL-8** | 2.76 (1.33-5.72), p=0.006 |
|  |  | **Periostin** | 2.72 (1.34-5.49), p=0.005 |
|  |  | **SP-D** | 2.60 (1.23-5.52), p=0.013 |
|  |  | **CCL18** | 2.38 (1.13-5.03), p=0.023 |
| Disease subtype | Diffuse Disease n=135 | **VCAM-1** | 3.72 (1.81-7.63), p<0.001 |
|  |  | **MMP-12** | 2.66 (1.18-6.00), p=0.018 |
|  |  | **MMP-1** | 2.40 (1.30-4.41), p=0.005 |
|  |  | **E-Selectin** | 2.31 (1.21-4.40), p=0.011 |
|  |  | **TIMP-1** | 2.19 (1.17-4.10), p=0.014 |
|  |  | **SP-D** | 2.19 (1.12-4.28), p=0.022 |
|  |  | **IL-6** | 2.16 (1.17-3.96), p=0.013 |
|  |  | **CXCL/IL-8** | 2.12 (1.13-3.99), p=0.020 |
|  |  | **CXCL13** | 2.02 (1.10-3.71), p=0.024 |
|  |  | **CXCL12** | 1.99 (1.07-3.71), p=0.030 |
|  |  | **CCL18** | 1.90 (1.03-3.52), p=0.041 |
|  |  | **CXCL4** | 1.85 (1.00-3.41), p=0.049 |
|  | Limited disease n=272 | **VCAM-1** | 3.68 (2.23-6.05), p<0.001 |
|  |  | **MMP-3** | 2.77 (1.75-4.38), p<0.001 |
|  |  | **IL-6** | 2.57 (1.63-4.06), p<0.001 |
|  |  | **MMP-7** | 2.42 (1.52-3.84), p<0.001 |
|  |  | **SP-D** | 2.41 (1.49-3.89), p<0.001 |
|  |  | **ET-1** | 2.35 (1.48-3.73), p<0.001 |
|  |  | **E-Selectin** | 2.15 (1.38-3.37), p=0.001 |
|  |  | **CXCL13** | 2.01 (1.29-3.15), p=0.002 |
|  |  | **CXCL10** | 1.97 (1.17-3.30), p=0.010 |
|  |  | **CXCL12** | 1.77 (1.13-2.76), p=0.012 |
|  |  | **Periostin** | 1.63 (1.05-2.55), p=0.031 |
|  |  | **CCL2** | 1.56 (1.00-2.44), p=0.049 |
| ILD extent | ILD extent <20% n=111 | **VCAM-1** | 3.64 (1.56-8.49), p=0.003 |
|  |  | **IL-6** | 2.69 (1.31-5.52), p=0.007 |
|  |  | **Periostin** | 2.62 (1.22-5.61), p=0.013 |
|  |  | **ET-1** | 2.57 (1.20-5.50), p=0.015 |
|  |  | **CXCL12** | 2.50 (1.20-5.18), p=0.014 |
|  |  | **E-Selectin** | 2.49 (1.21-5.14), p=0.014 |
|  |  | **MMP-3** | 2.30 (1.09-4.84), p=0.028 |
|  | ILD extent >20% n=86 | **SP-D** | 2.50 (1.37-4.56), p=0.003 |
|  |  | **CXCL12** | 2.24 (1.21-4.16), p=0.010 |
|  |  | **CCL18** | 2.19 (1.17-4.11), p=0.015 |
|  |  | **MMP-1** | 1.95 (1.05-3.65), p=0.035 |
| PAH | Yes n=70 | **SP-D** | 2.77 (1.36-5.66), p=0.005 |
|  |  | **E-Selectin** | 2.66 (1.40-5.04), p=0.003 |
|  |  | **VCAM-1** | 2.14 (1.18-3.90), p=0.013 |
|  |  | **Amphiregulin** | 1.98 (1.09-3.59), p=0.025 |
|  |  | **Ca15-3** | 1.89 (1.04-3.44), p=0.038 |
|  |  | **MMP-1** | 1.87 (1.02-3.42), p=0.043 |
|  | No n = 337 | **VCAM-1** | 3.54 (2.11-5.93), p<0.001 |
|  |  | **MMP-3** | 2.15 (1.35-3.42), p=0.001 |
|  |  | **IL-6** | 2.04 (1.29-3.21), p=0.002 |
|  |  | **CCL2** | 1.89 (1.17-3.04), p=0.009 |
|  |  | **CXCL12** | 1.85 (1.16-2.95), p=0.009 |
|  |  | **SP-D** | 1.84 (1.14-2.97), p=0.012 |
|  |  | **CCL18** | 1.82 (1.16-2.87), p=0.010 |
|  |  | **MMP-7** | 1.81 (1.14-2.86), p=0.012 |
|  |  | **ET-1** | 1.71 (1.09-2.70), p=0.020 |
|  |  | **CXCL13** | 1.64 (1.04-2.57), p=0.033 |
|  |  | **E-Selectin** | 1.64 (1.04-2.57), p=0.033 |
|  |  | **Periostin** | 1.58 (1.00-2.49), p=0.048 |

**Supplementary Table S2**. **Systemic sclerosis subgroup biomarker univariable analysis for mortality.**


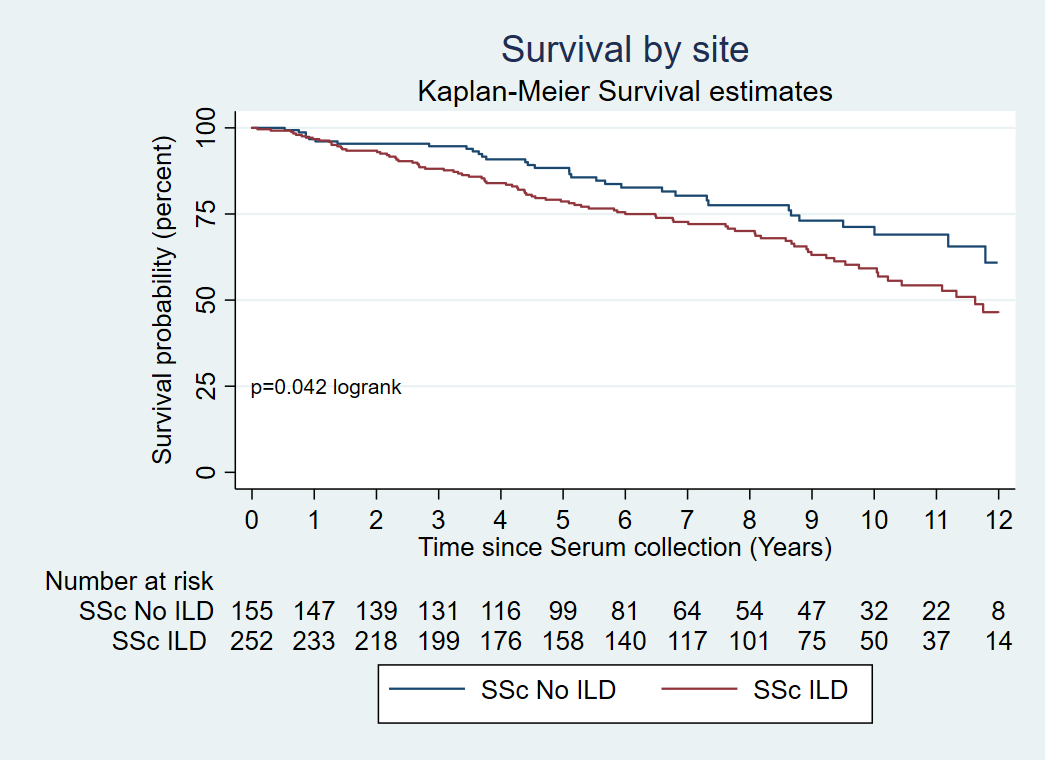


**Supplementary Figure S1: Survival probability in participants with and without ILD at baseline**
